# Assessing health system responsiveness in primary health care facilities in Tanzania

**DOI:** 10.1186/s12913-020-4961-9

**Published:** 2020-02-10

**Authors:** Ntuli A. Kapologwe, Stephen M. Kibusi, Josephine Borghi, Dorothy O. Gwajima, Albino Kalolo

**Affiliations:** 1 Department of Health, Social welfare and Nutrition Services, President’s Office Regional Administration and Local Government (PORALG), P. O Box 1923, Dodoma, Tanzania; 2 College of Health Sciences, School of Nursing and Public Health, University of Dodoma, P. O Box 395, Dodoma, Tanzania; 3 Department of Global Health and Development, London School of Hygiene & Tropical Medicine, 15-17 Tavistock Place, London, WC1H 9SH UK; 4 President’s Office Regional Administration and Local Government (PORALG), P. O Box 1923, Dodoma, Tanzania; 5 Department of Public Health, St. Francis University College of Health and Allied Sciences, P. O Box 175, Ifakara, Tanzania

**Keywords:** Health care delivery, Health system responsiveness, Primary health facilities, Tanzania

## Abstract

**Background:**

Health system performance is one of the important components of the health care delivery; its achievement depends on the quality of services rendered and the health system responsiveness of its beneficiaries. Health system responsiveness is a multi-dimensional concept and is usually measured through several domains. Health system responsiveness (HSR) remains to be a key indicator for evaluation of health system performance in any settings. This study aimed at assessing the situation of health system responsiveness in primary health facilities in Tanzania prior to introduction of the Direct Health Facility Financing (DHFF) program.

**Methods:**

This was a cross sectional study conducted between January and February in 2018. We collected data from 42 primary health facilities (14 health centers and 28 dispensaries) where a questionnaire was administered to a total of 422 participants. The questionnaire collected information on attention, respect to dignity, clear communication, autonomy, access to care, respect to confidentiality and basic amenities. Descriptive analysis was done to determine the distribution of the variables whereas ANOVA and linear regression analysis was employed to discern the association between variables.

**Results:**

More than 67% of participants had visited the same health facility more than 5 times. Sixty seven percent of the patients were residing within 5kms from the public primary health care facilities. The geographical access to health care scored the lowest (43.5% for Dispensaries and 36% for Health center) mean as compared to other domains of health system responsiveness. The highest score was in respect to confidentiality (86.7%) followed by respect to dignity (81.4%). Linear regression analysis revealed no statistical association between any of the social demographic features with the overall HSR performances. However, in post hoc analysis, Pwani and Shinyanga regions didn’t differ significantly in terms of their performances whereas those two regions differ from all other regions.

**Conclusion:**

Based on the study findings health system responsiveness domains has performed relatively poor in many regions except for respect of dignity and confidentiality scored high of all the domains. Shinyanga and Pwani regions scored relatively well in all domains this could have been due to the effect of Results Based financing (RBF) in the respective regions. All in all the Government and other stakeholders in the health sector they should deliberately invest on the access to care domain as seem to be a challenge as compared to others.

## Background

Well functioning health systems are critical for delivery of quality health services globally. The world health organization (WHO) has identified three intrinsic goals that are necessary for a health system to perform namely; improving health of the population, fairness in financial contribution and improving the responsiveness of the health system to the population it serves [1, 2]. There is a litany of evidence on the first two goals, but health system responsiveness (HSR) remains partially studied in low and middle income countries, therefore this study will offer an insight on the HSR performance in Tanzania.

Understanding Health System Responsiveness (HSR) is vital in the development of people centered health care systems, specifically so in the primary healthcare settings where majority (95%) of the patients access health services [1, 3].. Health system responsiveness entails the measures of the non-health aspect of care relating to the environment and the way healthcare services are provided to patients to meet their legitimate expectations. Responsiveness relates to a system’s ability to respond to the legitimate expectations of potential users or clients about non-health enhancing aspects of care and in broad terms [2]. Existing body of evidence in the HSR domain is limited to hospital settings and very scanty evidence exists in relation to the primary health care facilities [4–6]. Furthermore, most of the previous HSR assessments were done with a disease specific focus, such as heart failure, and among people enrolled into health insurance schemes [5, 7–9].

Health system Responsiveness can be used as a tool for evaluating the quality of health services rendered to the clients and offer feedback to both policy makers and implementers. HSR is multi-dimensional and mainly focuses on the seven domains namely: attention, autonomy, access to care, basic amenities of care, clear communication, confidentiality and respect to dignity [10]. The health system responsiveness depends mainly on the financial and social development as well as the capacity of the health care system; thus, there is a considerable difference between countries, developed countries faring better than low and middle-income countries [10].

Health system responsiveness has become a major consideration in assessment of the quality of any heath care system around the globe, where responsiveness has improved; other health outcome indicators were improving as well [11].

Evaluations of the performance of health systems across the globe have shown variation across countries with lower income countries lagging behind [12]. Some evidences from low and middle income countries shows that health system responsiveness in health care delivery tends to be ignored and many times not sufficient to meet patient’s non-medical demands [10]. Currently, there is a growing interest in evaluating people’s experience with health care services in Low and Middle-income countries so as to ascertain the level of satisfaction of patients to healthcare system [11–15]. In all settings, the individuals who are always and usually touched by poor responding health care system are women, as their demands for the health care system are wide and are multi-dimensional [16]. Women health is considered to be the litmus paper of a well functioning health system and gender power representation [17].

The health system responsiveness is not a well know concept and sometimes ignored in many countries, particularly in sub Saharan African (sSA) and some parts of Eastern Europe, specifically at the primary health care level [12]. In 2018, the Tanzanian government introduced a Direct Health Facility Financing (DHFF) reform with the idea that the reform will improve health system performance, in particular health system responsiveness and structural quality of services, especially of maternal and child health services.

**Fig. 1:**
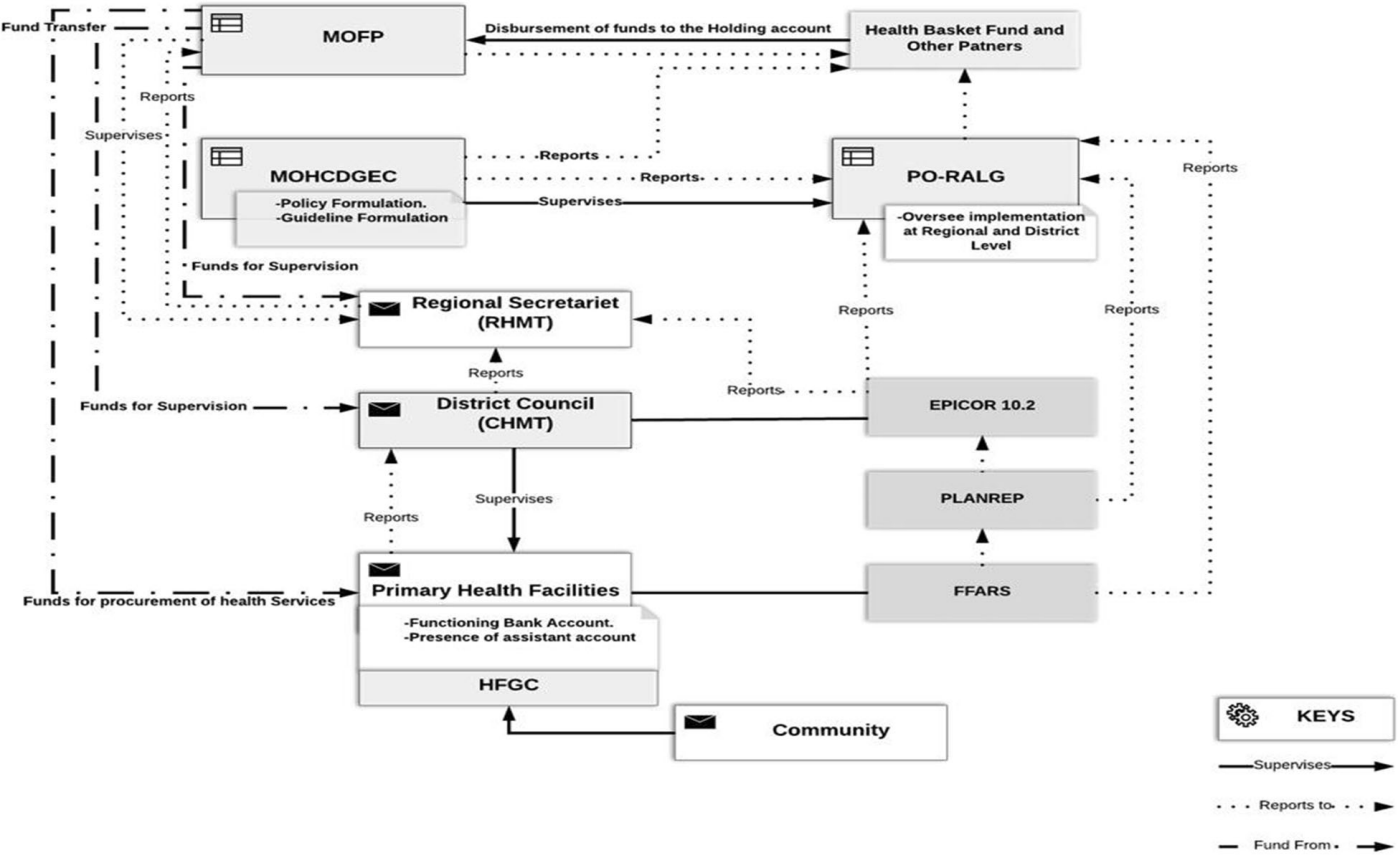
Direct Health facility Financing (DHFF) Funds flow and stakeholder’s relationship

The current study is part of the larger before and after evaluation study [18] that aims to establish the effects of the direct health facility financing reforms on health system responsiveness as described by Fig. 1 which shows the DHFF stakeholder’s interactions and funds flow. More specifically this study aimed to assess the status of health system responsiveness in primary health facilities prior to the implementation of the Direct Health Facility Financing (DHFF) program.

## Methods

### Study settings

In order to understand the Health system responsiveness in primary health facilities scattered in different geographical zones of the country, this study was conducted in seven regions namely Mbeya, Dodoma, Pwani, Shinyanga, Katavi, Manyara and Mtwara. The regions represent the seven zones of the country and comprise of 27% of the Tanzanian population. These regions were selected from respective zones so that to seek for generalizability of the study findings as there is geographical and cultural variation across the country. Approximately 70% of the Tanzanian population reside in rural areas and mainly depend on primary health facilities to address their health needs. The study included 42 health facilities (14 health centers and 28 dispensaries) located in 14 local government councils. The primary health facilities in Tanzania are divided into dispensaries and health centers. Dispensaries provide a basic range of preventive, health promotion, curative and maternal and child health (MCH) care, and health centres, offer inpatient and a higher level of delivery care and staffed by a wider range of more qualified health workers between 39 up to 52 than in the dispensaries which have between 15 up to 20 health service providers [19]. District hospitals serve as a referral at the primary health care level.

There are some of the programs being implemented in some selected regions such studies are: - Results Based Financing (RBF). RBF is defined as “a cash payment or non-monetary transfer made to a national or sub-national government, manager, provider, payer or consumer of health services after pre-defined results have been attained and verified. Payment is conditional on measurable actions being undertaken [20]. These payments are usually made to health providers after performance of the pre-defined results from selected quantitative and qualitative indicators. Before commence of the RBF implementation, each facility has to develop a business plan, which act as guide during the course of implementation. A Business Plan is a quarterly work plan of the facility, which shows the targets to be reached and strategies required to reach the targets through identified qualitative and quantitative indicators. It is a tool to help health facility staffs and stakeholders to develop their ideas and innovations to improve their efficiency.

### Study design

This study employed a cross sectional study design. The study was done between January and early February 2018.

### Sampling and sample size

Sampling was done using a four-stage sampling approach. The first stage included random selection of seven regions from the seven regions of Tanzania, located in seven geographical zones (each zone constituted between 3 and 4 regions). In the second stage, selection of district councils was done and two district councils basing on stratification were selected into one urban and one rural, in their respective regions. The third stage comprised of selection of health facilities to be included into the study, they were selected at random from each strata of each district council in the 7 regions. A total of 3 primary health facilities were selected randomly from each district’s list of each type of Public Primary Health Care Facilities (PPHCF) (i.e. 1 Health Centers and 2 Dispensaries) (http://hfrportal.ehealth.go.tz) [21] i.e. Making a total of 42 health facilities (14 health centers and 28 dispensaries).

The fourth stage included selection of the participants to participate in the study; the exit interview patients were conducted after they have received the services on their way to their homes. Respondents eligible for interview included all exiting patients or relatives of patients in case the patient is a minor (aged below 18 years), and stratified gender sampling was conducted to ensure that; there are an equal number of men and women in the study in order to ensure gender representation.

The sample size for patients/clients to participate in the study was determined by using the Cochran formula (1977) [22] with a 50.0% probability of the responsiveness of patients to primary health facilities, a α – error of 5%, an 80% power and a 95% confidence interval [23]. The calculation indicated a sample size of 384 with a 10% non-response contingency being added, making the total sample size required 422 patients. Each patient or relative of patients were systematically selected for an exit interviews after receiving medical consultations basing on their gender stratification. Ten patients were interviewed per each primary health care facility. Respondents eligible for interview included all exiting patients or relatives of patients (aged above18 years), and were sampled to ensure an equal number of men and women.

### Data collection tools and procedures

A closed-ended structured questionnaire was adapted from the health systems responsiveness questionnaires used in the WHO multi-country studies [4, 24, 25]. This standardized d questionnaire had 37 health system responsiveness closed-ended Likert scale questions that were grouped under 7 domains that have ordinal response categories [24]. The 37 questions were divided among the seven domains of responsiveness namely: prompt attention (7 questions), respect for dignity (3 questions), and communication (7 questions), quality of basic amenities (10 questions), respect to confidentiality (3 questions), access to care (4 questions) and autonomy (3 questions) (Additional file 1). The questionnaire was administered to systematically selected patients (10 patients per health facility) exiting the health facility, to measure their experiences with health care services.

To ensure accuracy of the collected information, research assistants underwent 4 days training on data collection using mobile phone devices (Tablets) before taking part in pre-testing of the questionnaires. All the questionnaires were then installed into the designed application in the mobile phone. All selected primary health facilities had GPS coordinates and all the data enumerators used tablets with GPS sensors so that to increase data integrity [26]. Mobile phone (Tablets) had a web-based interface that allows real-time gathering of data and the first author to monitor the data collection exercise on daily basis. After the actual field survey, collected data was then sent directly to the Gmail account app (which acted as a server) after being filtered in the field. For this study, the database (data collection software) was developed to which all the data obtained from the study units were entered. The patient survey data was captured on mobile phone then entered into a pre-designed database. The collected data were transferred into a Microsoft excel Database, and then exported to Statistical package for Social Sciences (SPSS) version 25 for statistical analysis. Data cleaning was undertaken before statistical analyses.

### Data analysis

#### Variables and their measurements

In this study socio-demographic and health system responsiveness variables were measured from the questionnaire as detailed below.

##### Socio demographic variables

These included; age, sex, educational status, marital status, number of visits to the primary health care facilities, distance covered by a patient to get the health facility, and family size. In this study, age was measured in years, sex was categorized into male or female and marital status was categorized into single or married. We measured distance covered by a patient to access health care in kilometres whereas family size was measured by number of household members and number of health facility visitation was measured in days (Table 1).

**Table 1:** Respondents’ Social Demographic characteristics (*n* = 422)

Characteristic	Rural	Urban
	N (%)	N (%)
Gender of the participants		
Male patient	103 (24.5)	106 (25.2)
Female patient	105 (25.0)	106 (25.2)
Ages of the participants		
15–24 years	45 (10.7)	35 (8.3)
25–35 years	73 (17.4)	77 (18.3)
36–44 years	46 (11.0)	44 (10.5)
45 and above	44 (10.5)	56 (13.3)
Marital status		
Married	164 (39.1)	170 (40.5)
Single	39 (9.3)	38 (9.0)
Widow/widowed	5 (1.2)	4 (1.0)
Education level		
Primary	133 (31.7)	132 (31.4)
Secondary	26 (6.2)	43 (10.2)
Post Secondary	49 (11.6)	28 (9.8)
What is the size of your family?		
Below or 3 members	57 (13.6)	60 (14.3)
4–6 members	82 (19.5)	99 (23.6)
Above 6 members	69 (16.4)	53 (12.6)
Patient’s number of visits to the facility before		
Twice	24 (5.7)	27 (6.4)
Thrice	15 (3.6)	26 (6.2)
Four times	20 (4.8)	13 (3.1)
Five times	17 (4.0)	11 (2.6)
More than five times	132 (31.4)	135 (32.1)
Distance covered to get health services		
Within 5 km	144 (34.3)	138 (32.9)
5–10 km	42 (10.0)	47 (11.2)
Above 10 km	22 (5.2)	27 (6.4)

##### Health system responsiveness (HRS)

Measures the non-health aspect of care relating to the environment and the way healthcare services are provided and relates to a system’s ability to respond to the legitimate expectations of potential users or clients. In this study health system Responsiveness mainly focused on the seven domains that are: attention, autonomy, and basic amenities of care, access to care, clear communication, confidentiality, and respect of dignity. All questions were Likert scale in nature and grouped under seven domains. Each domain was measured by using a mean score and then they were compared among health facilities and among regions (Table 2). The internal consistency reliability of the overall responsiveness scale (37 items) was calculated and average Cronbach’s alpha for all seven domains was 0.827.

**Table 2:** Health care responsiveness performance criteria and their Categorization

Domain	Number of questions	Min-max score(s)	Unacceptable	Acceptable	
			Fail (%)	Good (%)	Very Good (%)
Prompt attention	7	0–21	0.0–33.3	33.4–66.7	66.8–100
Respect for dignity	3	0–9	0.0–33.3	33.4–66.7	66.8–100
Clear communication	7	0–21	0.0–33.3	33.4–66.7	66.8–100
Respect of autonomy	3	0–9	0.0–33.3	33.4–66.7	66.8–100
Access to care	4	4–16	0.0–25.0	25.1–50.0	50.1–100
Respect for confidentiality	3	0–9	0.0–33.3	33.4–66.7	66.8–100
Quality of basic amenities	10	10–40	25.0–50.0	50.1–75.0	75.1–100
Overall responsiveness	37	14–125	0.0–29.9	30.0–59.2	59.3–100

#### Statistical analyses

The first step did include conducting descriptive statistics (frequency, percentage, mean and standard deviation) analysis of the health system responsiveness of all seven domains. Analyses of health system responsiveness scores for all variables of socio demographic were conducted.

The Health System Responsiveness was analyzed basing on the primary health facilities user’s experiences as shown in the four points Likert scales. Each point of the Likert scale was in the percentage and the answers were then dichotomized for further analysis for example good and very good as ‘Good’ and bad and very bad as ‘Bad’. The Likert scale rating for each domain was matched with the responsiveness performance categories as ‘unacceptable’ (Fail) and ‘acceptable’ (Good and Very Good) (Table 2). For instance, the corresponding code for response for basic amenities domain was four that was multiplied by ten (the number of questions in the domain) that produced a cut-off score of 40 that makes a maximum score (“Acceptable”) for an individual for this Domain, and one multiplied by 10 (number of questions) making minimum score (“Fail”) of 10 for each individual who responds to all questions for this Domain (Table 2). This approach is similar to that was used in another study conducted in Ethiopia in 2017 [31].

A total of 37 questions were included to assess health system responsiveness in the primary health care facilities in Tanzania. Four points Likert scales question items ranging from 0 to 3 for five domains (attention, dignity, communication, autonomy and confidentiality) in which 0 represented absence of the assessed feature of HSR and 3 denoting the highest level of its availability. On the other hand, 1 to 4 points were used for two domains (access to care and quality of basic amenities) with 1 score indicating the least performance of the assessed HSR feature and 4 for the highest level of the availability of the features. In total, a minimum of HSR score was computed as 14 out of the maximum score of 125 for all 37 questions (Table 2). Total score for each domain was computed in percentage by taking the actual score obtained from each respondent divided by the maximum possible score multiplied by 100%. Similarly, the overall HSR score was computed by dividing the overall total scores over the maximum possible value (125) multiplied by 100%.

The second step was to conduct the inferential statistics. In order to conduct some inferential statistics, the basic assumptions for normality test were conducted. Visual inspection of histogram Q-Q and Box plots (graphs) was done. In addition, skewness and kurtosis z-values and Shapiro-Wilk test for dependent variables were also conducted. Visual inspection of histograms indicated that dependent data distribution were along the straight line for Q-Q plots for both dispensary and health centre whereas symmetrical feature was observed on box plots for both dispensary and health centre. Shapiro Wilk test showed *p* ≥ .005 (*p* = .694 and .828 for dispensary and health centre, respectively).

Skewness and kurtosis z-values were within the range of − 1.96 to + 1.96 for both dispensary and health centre (dispensary = −.0599, .543 and health centre = −.245, .543). All this suggests that data were approximately reasonably normally distributed. Therefore, parametric tests for inferential statistics were considered relevant for performance comparison.

##### Analysis of variance (ANOVA)

Was used to compare the means of more than two groups especially the regional level comparisons that has tried to display the mean of each region allowing for comparison of overall performance on different assessed aspects (domains).

##### Multiple regression analysis

Was used to explore the predictor power of each independent variable on a dependent variables specifically demographic information and perceived responsiveness. It was also used to assess the power of predictors for institutional factors (e.g. population, staffing level, number of beds etc) with the perceived responsiveness.

## Results

### Socio-demographic characteristics

A total of 422 patients participated in the study of which 50% were female. About 72% of all study participants were married with 23% of them having an average of 6 members per household. More than 67% of patients were the ones who have had more than 5 times visits to the health care facility. About 35.5% of patients had age of between 25 and 35 years. The 63% of participants had a secondary school education. Majority (68.9%) of Health Centers has less than 39 skilled staff while some of them they have up to 129 and majority (92.8%) of Dispensaries have less than 15 staff and some of them have 1 staff (Table 1).

### Health system responsiveness (HSR)

#### Scores of the seven domains of healthcare system responsiveness

Looking into the performance score of all seven domains of health system responsiveness, access to care had least performance of below 50% while other domain performed relatively higher than 50% with confidentiality revealing an outstanding performance for both dispensaries (86.5%) and health centers (90.2%) respectively. Generally, performance on HSR indicated that the highest scores were recorded on respect for confidentiality while the least performing domain was access to care. Of all the health system responsiveness domains, access to care (43.5% for Dispensaries and 36.0% for Health Center) had low ‘unacceptable/ fail’ health system responsiveness while respect to confidentiality (86.7% for Dispensaries and 90.7% for Health center) high ‘very good’ health system responsiveness (Tables 2 & 3).

**Table 3:** Means comparison on dispensary and health centre (*N* = 422)

HSR domains	Dispensary		Health centre		Sig.	Eta squared
	Mean	SD	Mean	SD		
Total HSR %	69.6	11.7	68.0	11.3	.189	.004
Prompt attention %	78.6	20.9	74.1	21.8	.042	.010
Respect for dignity %	81.4	20.9	76.5	25.3	.037	.010
Clear communication %	74.3	24.8	73.9	24.7	.897	.000
Respect for Autonomy %	76.7	26.9	72.1	28.6	.107	.006
Access to care %	43.5	12.5	36.0	10.9	.000	.077
Confidentiality %	86.7	29.8	90.7	20.0	.157	.005
Quality of Basic Amenities %	64.6	12.6	66.4	10.5	.161	.005

**Fig. 2:**
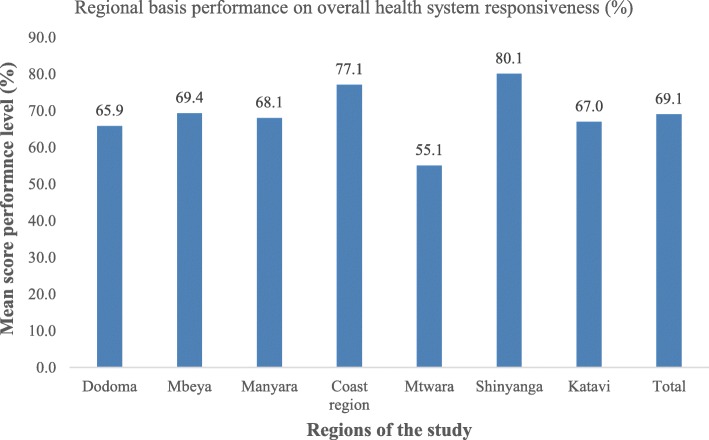
Overall health system responsiveness performance by regions

From Fig. 2 it is noted that; Shinyanga and Coast region outperformed all other regions in all seven domains. On the other hand, Mtwara demonstrated the lowest performance as compared to other regions. On the other hand, Mtwara demonstrated the lowest performance as compared to other regions, with Dodoma (65.9%), and Katavi (67.0%) who also fell below the mean percentage that was 69.1% [67.9–70.2] (Table 2).

Analysis of variance (ANOVA) for means comparison indicated marginal difference between dispensary and health centre on different domains. Statistical significant differences were observed at prompt attention (*p*=. 042), dignity (.037) and access to care (*p* < .0005). Generally, health centre and dispensary did not differ significantly on overall HSR performance and other domains namely, clear communication (*p*=. 897), autonomy (.107), confidentiality (.157) and quality of basic amenities (.161). The magnitude of differences on Eta squared indicated very small effect on differences among the domains with highest value indicated at access to care (.077) and the lowest at communication (Table 3).

#### Factors associated with HSR

Linear regression analysis was conducted to assess factors associated with HSR. It was indicated that there was no statistical correlation coefficients between any of the social demographic features with the overall HSR performances (Table 4). The highest performing regions were Pwani and Shinyanga regions while the least performing region was Mtwara. Analysis of variance indicated significant differences between Shinyanga, Coast Region and Mtwara as compared to all other regions. Generally, Coast and Shinyanga regions did not differ significantly as indicated in the Table 5. Post hoc analysis indicates that, Coast regions and Shinyanga do not differ significantly in terms of performances, whereas these two regions differ significantly (*P* < .005) from all other regions included in the study.

**Table 4:** Linear regression analysis on health system responsiveness and covariates

Variables	Pearson Correlation	Sig. (1-tailed)
Total HSR %	1.000	.000
Type of health facility	−.064	.095
Gender of the participants	.010	.420
Ages of the participants	.042	.195
Marital status	−.090	.032
Highest level of education	.071	.072
Family size	.098	.022
Patient’s number of visits to the health facility before	−.024	.313
Distance covered by a patient to get health service	.103	.017

**Table 5:** Multiple Comparisons of Regions across overall performance on HSR

(I) Region	(J) Regions	Mean Difference (I-J)	Std. Error	Significance (95%CI)
Dodoma	Mbeya	−3.5024	1.6201	.319 (−8.302, 1.298)
	Manyara	−2.1957	1.6201	.825 (−6.996, 2.604)
	Coast region	−11.2624^a^	1.6201	.000 (−16.062, − 6.462)
	Mtwara	10.7675^a^	1.6342	.000 (5.926, 15.609)
	Shinyanga	−14.2065^a^	1.6067	.000 (−18.967, −9.446)
	Katavi	−1.1557	1.6201	.992 (− 5.956, 3.644)
Mbeya	Dodoma	3.5024	1.6201	.319 (−1.298, 8.302)
	Manyara	1.3067	1.6333	.985 (−3.533, 6.146)
	Coast region	−7.7600^a^	1.6333	.000 (−12.599, −2.921)
	Mtwara	14.2699^a^	1.6473	.000 (9.389, 19.151)
	Shinyanga	−10.7041^a^	1.6201	.000 (−15.504, −5.904)
	Katavi	2.3467	1.6333	.782 (−2.493, 7.186)
Manyara	Dodoma	2.1957	1.6201	.825 (−2.604, 6.996)
	Mbeya	−1.3067	1.6333	.985 (−6.146, 3.533)
	Coast region	−9.0667^a^	1.6333	.000 (−13.906, −4.227)
	Mtwara	12.9632^a^	1.6473	.000 (8.082, 17.844)
	Shinyanga	−12.0108^a^	1.6201	.000 (−16.811, −7.211)
	Katavi	1.0400	1.6333	.996 (−3.799, 5.879)
Coast region	Dodoma	11.2624^a^	1.6201	.000 (6.462, 16.062)
	Mbeya	7.7600^a^	1.6333	.000 (2.921,12.599)
	Manyara	9.0667^a^	1.6333	.000 (4.227, 13.906)
	Mtwara	22.0299^a^	1.6473	.000 (17.149, 26.911)
	Shinyanga	−2.9441	1.6201	.537 (−7.744, 1.856)
	Katavi	10.1067^a^	1.6333	.000 (5.267,14.946)
Mtwara	Dodoma	−10.7675^a^	1.6342	.000 (−15.609, −5.926)
	Mbeya	−14.2699^a^	1.6473	.000 (−19.151, −9.389)
	Manyara	−12.9632^a^	1.6473	.000 (−17.844, −8.082)
	Coast region	−22.0299^a^	1.6473	.000 (−26.911, −17.149)
	Shinyanga	−24.9740^a^	1.6342	.000 (−29.816, − 20.132)
	Katavi	−11.9232^a^	1.6473	.000 (−16.804, −7.042)
Shinyanga	Dodoma	14.2065^a^	1.6067	.000 (9.446, 18.967)
	Mbeya	10.7041^a^	1.6201	.000 (5.904, 15.504)
	Manyara	12.0108^a^	1.6201	.000 (7.211, 16.811)
	Coast region	2.9441	1.6201	.537 (−1.856, 7.744)
	Mtwara	24.9740^a^	1.6342	.000 (20.132, 29.816)
	Katavi	13.0508^a^	1.6201	.000 (8.251, 17.851)
Katavi	Dodoma	1.1557	1.6201	.992 (−3.644, 5.956)
	Mbeya	−2.3467	1.6333	.782 (−7.186, 2.493)
	Manyara	−1.0400	1.6333	.996 (−5.879, 3.799)
	Coast region	−10.1067^a^	1.6333	.000 (−14.946, − 5.267)
	Mtwara	11.9232^a^	1.6473	.000 (7.042, 16.804)
	Shinyanga	−13.0508^a^	1.6201	.000(−17.851, −8.251)

^a^ The mean difference is significant at the 0.05 levels

## Discussion

This study aimed at assessing the situation of health system responsiveness prior to the inception of DHFF program in Tanzania. In this study, of all the seven domains which were assessed, respect for confidentiality (87.9%), Dignity (79.9%) and prompt to Attention (77.2%) had high score whereas Basic amenities (65.2%) and access to care (41.2%) had the lowest score.

In this study it was clear that majority of the regions had just acceptable scores in terms of their responsiveness in relation to the maternal health services except for two regions namely Shinyanga and Pwani which might have been due to the already existing results based financing (RBF) program.

The results from this study showed that none of the social demographic characteristics were significantly associated with the Responsiveness percentage Score. However, one study from Nigeria have shown that type of facility, gender, education status, marital statuses were strongly associated with health system responsiveness [26].

Responsiveness being the ability to respond to the legitimate expectations of potential users about non-health enhancing aspects of care, in this study we found that, of the seven domains which were measured, confidentiality and clear communication had high mean scores; with regard to confidentiality this might have been due to the ongoing primary health care development program since 2007 where the primary health care facilities are the main target for infrastructural improvement, this program has been coupled with provision of training, workshops and offering of Standard Operating Procedures (SOP) on confidentiality and effective communication at the primary health facilities levels, the findings of this study is in line with other studies which were conducted in Ethiopia and Iran [27, 28]. While contrarily to another study done in China whereby dignity and confidentiality were ranked highest while choice and prompt attention lowest [29]. However, ratings of health care system responsiveness differ across different studies [8].

Findings from this study didn’t show if the HSR was affected by geographical location (rural vs. urban) while studies in China has shown that [29] geographical location do affect the HSR.

As it can be noted from the findings, Shinyanga and coast regions outperformed all other regions in all domains with exception of access to care domain in which two regions performed relatively lower than Dodoma and Katavi. Furthermore, overall performances also depicted higher performance of the two regions on the total responsiveness this could probably be due to effect of the RBF program which currently is implemented in 7 regions including Shinyanga and Pwani, among other things under the RBF program arrangement it supports certain indicators like improvement of amenities and incentive and motivation for the health service providers [30].

From the finding of this study, it is clearly shown that; two domains namely basic amenities and Confidentiality performed relatively low in Dispensaries as compared to the Health Centers this could be explained probably by availability of more spacious building with many functionality in health centers as compared to Dispensaries.

Health system responsiveness in Tanzania is of great importance as the country has gone through so many reforms in the health sector which are worth while being assessed its effects to the beneficiaries. Therefore, understanding the health system responsiveness which when they are improved have had an effect on others indicators of the health care system. This can also help to address the challenges around respective maternity care that has reported in some studies in Tanzania [30].

Despite the fact that, it is the first time Health system responsiveness assessment study being conducted at the primary health care level in Tanzania. This study is limited, because study participants self reported their experiences, which may lead into, bias and also it was a cross sectional study which is good to get snap shot findings.

## Conclusion

Based on the study findings health system responsiveness domains has performed relatively poor in many regions except for respect of dignity and confidentiality scored high of all the domains. Shinyanga and Pwani regions scored relatively well in all domains this could have been due to the effect of Results Based financing (RBF) in the respective regions. All in all the Government and other stakeholders in the health sector they should deliberately invest on the access to care domain as seem to be a challenge as compared to others.

## Supplementary information

**Additional file 1.** Questionnaire on patient exit interview.

## Data Availability

It is declared that data and materials are available. They can be sought from the corresponding author (first author).

## Abbreviations

CHMT: Council Health Management Team
DHIS-2: District Health Information System − 2
DMO: District Medical Officer
HFGC: Health Facility Governing Committee
HMIS: Health Management Information System
HSR: Health System Responsiveness
MoHCDGEC: Ministry of Health Community Development, Gender, Elderly and Children
PO-RALG: President’s Office – Regional Administration and Local Government
PPHCF: Public Primary Health Care Facilities
RAS: Regional Administrative Secretary
RBF: Results Based Financing
RHMT: Regional Health Management Team
