# Supplementary material for: Assessing health system responsiveness in primary health care facilities in Tanzania
Source: BMC Health Serv Res. 2020 Feb 10;20:104. doi: 10.1186/s12913-020-4961-9 (PMC7011252; doi:10.1186/s12913-020-4961-9)
Supplement: Supplementary file 1 — Additional file 1. Questionnaire on patient exit interview. [file 12913_2020_4961_MOESM1_ESM.docx]

**Questionnaire for patient exit interview**

***Kiambatanisho na.3 Dodoso la mgonjwa aliyemaliza huduma kituoni.***

Objective 3 (Impact assessment): To assess the effect of the DHFF program on responsiveness to the health system as perceived by the end users.

***Lengo Na.3 (Uhakiki wa matokeo): Kuhakiki matokeo ya mfumo wa kutoa fedha kwenye vituo moja moja kwenye mapokeo ya mfumo wa huduma za afya kwa wananchi.***

Serial No. [__|__|__|__]

***Namba ya kumbukumbu***

Name of the Region: _____________________________________

***Jina la Mkoa;***

Name of the District Council: ______________________________

***Jina la Wilaya:***

Ward _____________________________Village/Street _______________

***Kata ______________________________ Kijiji/ Mtaa________________***

Name of the Health Facility: _______________________________

***Jina la kituo:***

Type of Health Facility: 01= Dispensary [ ] 02= Health center [ ]

***Aina ya kituo: 01 = Zahanati [ ] 02 = Kituo cha afya [ ]***

Please put the appropriate number of a response in the given box

***Tafadhali weka namba ya jibu sahihi kwenye chumba ulichopewa***

| SN  ***Na.*** | Questions  ***Maswali*** | | Responses  ***Majibu*** | Code | Code |
| --- | --- | --- | --- | --- | --- |
| SECTION A: DEMOGRAPHIC INFORMATION  ***KIPENGELE A: TAARIFA ZA AWALI ZA MDODOSWAJI.*** | | | | | |
|  | (a)Sex  ***Jinsia*** | 1.Male  ***Mwanaume***  2.Female  ***Mwanamke*** | | 1  0 | [ ] |
|  | (b)How old are you?  ***Una umri gani?*** | 1. 15 – 24 years 2. 25 – 35 year 3. 36 – 44 year 4. 45 and above | | 1  2  3  4 |  |
|  | Marital status  ***Hali ya ndoa*** | 1.Married  ***Nina ndoa***  2.Cohabiting  ***Tunaishi pamoja***  3.Single  ***Sina ndoa***  4.Divorced/Separated  ***Mtalaka***  5.Widow/widowed  ***Mjane / Mgane*** | | 01  02  03  04  05 | [ ] |
|  | Highest level of education  ***Kiwango cha elimu*** | 1.Primary  ***Msingi***  2.Secondary  ***Sekondari***  3.Certificate  ***Astashahada***  4.Diploma  ***Stashahada***  5.Advanced diploma  ***Astashahada ya juu***  6.University degree  ***Shahada***  7.Masters  ***Shahada ya uzamili***  8. Others  ***Nyingine*** | | 1  2  3  4  5  6  7  8 | [ ] |
|  | What is the size of your family?  ***Familia yako inawatu wangapi?*** | 1.Below 3 members  ***Chini ya watu watatu***  2. 4 – 6 members  ***Watu 4 mpaka 6.***  3. Above 6  ***Zaidi ya watu 6*** | | 1  2  3 |  |
|  | What is the number of visits you have had to this facility before?  ***Umewahi kupata huduma mara ngapi katika kituo hiki?*** | 1.Twice  ***Mara mbili***  2.Thrice  ***Mara tatu***  3.Four times  ***Mara nne***  4.Five times  ***Mara tano***  5. More than five  ***Zaidi ya mara tano*** | | 01  02  03  04  05 | [ ] |
|  | How much distance would you have to undertake in order to reach health care?  ***Unatembea umbali gani kuifikia huduma ya afya?*** | 1. Within 5 kilometers   ***Ndani ya kilomita 5***   1. 5 – 10 kilometers   ***Kilomita 5 mpaka 10***   1. Above 10 kilometers   ***Juu ya kilomita 10*** | | 1  2  3 |  |

| Prompt to Attention (7)  ***Umakini*** | Very often (3)  ***Mara zote*** | Often (2)  ***Mara kwa mara*** | Slightly Often (1)  ***Mara chache*** | Never happens (0)  ***Haijawahi kutokea*** |
| --- | --- | --- | --- | --- |
| 1. How often did the health service providers listen to what you said with full attention during provision?   ***Nimara ngapi watoa huduma wa afya wamekusikiliza kwa umakini wakati wa kupata huduma?*** |  |  |  |  |
| 1. How often your statements were deeply understood by the health service providers?   ***Nimara ngapi maeezo yako yameeleweka vyema kwa watoa huduma wa afya wa kituo hiki?*** |  |  |  |  |
| 1. How often did health service providers spend enough time in asking you questions?   ***Mara ngapi mtoa huduma ametumia muda wa kutosha kukuuliza maswali juu ya ugojwa wako?*** |  |  |  |  |
| 1. How often the health service providers were accurately and actively in following up your treatment process?   ***Ni kwa kiwango gani watoa huduma wa afya wamekuwa makini katika kufuatilia matibabu yako ndani ya kituo cha kutoa huduma?*** |  |  |  |  |
| 5. The patients with similar needs are treated equally in the health units? |  |  |  |  |
| 6.Patients with un equal needs are treated equally in the health units? |  |  |  |  |
| 7.The health has always met my expectations |  |  |  |  |
| Respect to Dignity (3)  ***Utu*** | | | | |
| 1.How often did the health service providers show courtesy and affection towards you during service provision?  ***Ni mara ngapi watoa huduma wa afya wameonyesha heshima na upendo kwako wakati wa kupata huduma?*** |  |  |  |  |
| 2.How often did the health care workers paid attention specifically into your needs and characteristics?  ***Ni mara ngapi mtoa huduma amekuwa makini hasa kwa mahitaji yako na hali yako?*** |  |  |  |  |
| 3.How often is respect shown for the patient’s desire for privacy during treatment and examination? |  |  |  |  |
| Clear Communication (7)  ***Mawasiliano bayana*** | | | | |
| 1.How often did health care workers explain things in a way you could understand?  ***Ni mara ngapi watoa huduma wameeleza vyema mambo yanayokuhusu katika njia unayoielewa?*** |  |  |  |  |
| 2.How often health care workers explain things and issues related to your health in detail for you?  ***Ni kwa kiwango gani watoa huduma wa afya hueleza hali yako ya afya kiundani kwako?*** |  |  |  |  |
| 3.How would you rate your experience about how well you were treated as human during interection with the following service providers?  Nurses |  |  |  |  |
| 1. Laboratory staff |  |  |  |  |
| 1. Medical doctors/clinicians |  |  |  |  |
| 1. Security staff |  |  |  |  |
| 1. How wiould overall rate quality of interection at this health facility? |  |  |  |  |
| Autonomy (2)  ***Uhuru wa kufanya mahamuzi*** | No problem ***(3)***  ***Hakuna tatizo*** | Average  ***(2)***  ***Wastani*** | Big ***(1)***  ***Kubwa*** | Very big  ***(0)***  ***Kubwa sana*** |
| 1.How big a problem if any was it to get an appointment with the health care worker of your choice?  ***Ni kwa kiwango gani ulipata tatizo (kama lipo) la kupata fursa ya kukutana na mtuo huduma wa afya uliyemhitaji?*** |  |  |  |  |
| 2.How big a problem if any was it to use other health facility other than the one you usually went to?  ***Nikwa kiwango gani ulipata tatizo (kama lipo) kutumia kituo kingine cha huduma ya afya zaidi ya kile ambacho huenda kila wakati?*** |  |  |  |  |
| Access to Care (4)  ***Upatikanaji wa huduma*** | Waited for long time (above 30min)  (1)  ***Nilisubiri muda mrefu (zaidi ya dakika 30)*** | Average (up to 30min)  (2)  ***Wastani (mpaka dakika 30)*** | Waited for few minutes  (3)  ***Kusubiri kwa dakika chache*** | Serviced instantly  (4)  ***Nilipata huduma mara moja*** |
| 1.How long did you have to wait to get medical consultation from service provider?  ***Ulitumia muda gani kusubiri huduma ya afya?*** |  |  |  |  |
| 2.How long did you have to stay in the waiting room?  ***Ulisubiri muda gani katika chumba cha kusibiri kumuona mtoa huduma wa afya?*** |  |  |  |  |
| 3.How long did you have to stay at the pharmacy or dispensing area? |  |  |  |  |
| 4.How long did you have to stay waiting for laboratory services and results? |  |  |  |  |
| Confidentiality (3)  ***Usiri*** | | | | |
| 1.How often interviews remained confidential?  ***Kwa kiwango gani mahojiano yamekuwa ya usiri?*** |  |  |  |  |
| 2.Health care workers keep your personal information and records confidential?  ***Watoa huduma ya afya huweka taarifa zako binafsi kwa siri?*** |  |  |  |  |
| 3.Is the confidentiality maintained in this health facility? |  |  |  |  |
| **Basic Amenities (11)**  ***Mahitaji muhimu*** | Strongly agree  (4)  ***Nakubali kabisa*** | Agree  (3)  ***Nakubali*** | Disagree  (2)  ***Sikubali*** | Strongly disagree  (1)  ***Sikubali kabisa*** |
| 1.I agree that this facility have enough buildings for service delivery  ***Nakubali kuwa kituo kina majengo ya kutosha ya kutoa huduma*** |  |  |  |  |
| 2.I agree that this facility have enough staffs to service patients  ***Nakubali kuwa hiki kituo kina watoa huduma wa kutosha kutoa huduma kwa wagonjwa*** |  |  |  |  |
| 3.I agree with the quality of direction aids of this facility  ***Nakubaliana na ubora wa vielelezo na miongozo iliyo kwenye kituo hiki.*** |  |  |  |  |
| 4.I agree with the cleanliness of this surroundings  ***Nakubaliana na hali ya usafi wa hiki kituo*** |  |  |  |  |
| 5.I agree with the waiting environment of this facility (waiting seats)  ***Nakubaliana na mazingira ya kusubiria huduma ya kituo hiki (ikiwa ni pamoja na viti)*** |  |  |  |  |
| 6.Are you convenient with the safety of service delivery environment in this facility?  ***Je unaridhishwa na mazingira ya usalama ya utoaji huduma wa hiki kituo?*** |  |  |  |  |
| 7. Access to clean water at health care units |  |  |  |  |
| 8. Cleaness of the toilets in the health care units |  |  |  |  |
| 9.Facilities for people with disabilities in the health care units |  |  |  |  |
| 10. The smell in the health care units |  |  |  |  |
| 11. Access to the soap at all hand washing areas |  |  |  |  |
